# Evaluating the institutionalisation of diversity outreach in top universities worldwide

**DOI:** 10.1371/journal.pone.0219525

**Published:** 2019-07-24

**Authors:** Mariana Buenestado-Fernández, José Luis Álvarez-Castillo, Hugo González-González, Luis Espino-Díaz

**Affiliations:** Department of Education, Faculty of Education Sciences, University of Cordoba, Cordoba, Spain; Università degli Studi di Perugia, ITALY

## Abstract

The participation of diverse demographics in higher education has risen over the last half-century; meanwhile, different political and social tiers have been assigning a more active role to institutions in terms of equality and social justice. This change in circumstances has led to the roll out of processes to institutionalise diversity outreach. This study was conducted for the clear purpose of assessing the current institutionalisation status of diversity outreach in 127 key universities from the Academic Ranking of World Universities based on the opinions of diversity outreach managers and the information published on institutional websites, in turn measuring compliance with various indicators. A qualitative analysis of the institutional statements, the goals sought through strategic plans and the definitions of diversity itself was also conducted. The evidence reveals the early stage of the institutionalisation process in universities on account of the low percentage obtained for the proposed indicators. Furthermore, the study failed to exhibit significant differences in this process in terms of the institutional ownership or position held in the ranking; however, more prominent progress was noted in the North-American region when geographical differences were taken into account, likely as a result of the historical background in the advocacy for equal opportunities. Lastly, a change of approach to the conceptualisation of diversity is suggested in favour of equality and social justice.

## Introduction

In the last half-century, higher education has been considered a key factor in economic prosperity and social well-being thanks to the dissemination of knowledge in the interest of securing sustainable human development [1]. In this regard, the radical expansion of higher education systems worldwide has led to the participation of larger demographics, even in low-income countries [2]. Thus, in more advanced societies, globalisation, the demographic shift and accomplishments in social justice have expanded the scope in which people differ within organisations in relation to how it was only several decades ago [3]. In reference to the traditional approach to diversity that registers primary factors including race or ethnicity, gender or age [4], it is clear how the differences are increasingly more prominent in universities: the number of students from ethnic minorities who embark on higher education studies progressively increases, such as the case of indigenous peoples in Latin American countries, whose participation rises 1% annually [5], or the number of black and Hispanic students in the United States, which increased from 10% to 14% and from 4% to 17% respectively between 1976 and 2014 [6]; the enrolment rate of women in higher education institutions has increased twice as more as that of men in the last four decades [7]; and an increase of 26% to 35% is estimated for the 25–64 year old demographic in higher education between 2005 and 2025 in OECD countries on the whole due to the promotion of lifelong learning and the increase in life expectancy [8].

### Comparative higher education in terms of diversity regulation

The inclusion of diversity in development policies and plans in higher education varies depending on the context or region of the world. In the United States, the mitigation of differences dates back to the Civil Rights Movement of the 1960s [9] with the approval of important regulations—i.e., the Civil Rights Act in 1964, and executive orders about affirmative action—, or even to previous steps, as the passage of the 14^th^ and 15th amendments to the Constitution in 1868 and 1870, respectively, or the 2^nd^ Morril Act that promoted the creation of the Historically Black Colleges and Universities in 1890 [10]. As a result of this sociological phenomenon, programs were put in place seeking enforcement of these rights on universities campuses [11, 12]. Although diversity has historical roots in equality policies, especially those aimed at race and ethnic minorities, American and Canadian universities have integrated other differences that lead to inequalities in their endeavours, such as the socio-economic status or educational level of parents, thus promoting the term 'inclusive excellence' [13].

In the European context, the reference to equal opportunities or social justice stems from transnational policy guidance. The European higher education policies developed a social dimension following the initial developments of the European Higher Education Area in 1998, which called for collective access, placing an emphasis on groups that had been under-represented thus far. In particular, gender and disability are the criteria for heterogeneity on which inclusive efforts have largely focused [14], although the European Commission, that promotes equity and inclusion through the Erasmus+ Programme, also considers criteria such as educational difficulties, economic limitations, cultural differences, health problems, social barriers and geographical obstacles [15]. Thus, as a result of a project co-funded by the European Commission, Dovigo and Casanova [16] report a collection of institutional policies and good practices carried out in higher education systems and universities of six European and four non-European countries. The considerable array of practices in a good number of domains and groups (e.g., scholarships for asylum seekers, curricular adaptations and peer tutoring services for students with disabilities, support services for parent students, promotion of volunteering and active solidarity, financial support for students from low income backgrounds, strategies to ensure access to the university for disadvantaged students, training programmes in inclusion) suggests that the culture of diversity could be spreading over Europe.

Likewise, the *Tuning* project [17], which is also rolled out in Latin America, considers a paradigm focused on students and highlights the celebration of diversity as a generic competence. In Latin American countries, civil society organizations have played an important role in promoting public policies on inclusive education in higher education. Groups and sectors of the original population that have historically been excluded and marginalized from education systems have organized themselves into movements (black movements, landless peasants, indigenous communities) to claim diversity as an expression of humanity and to fight for changes in social models and models of inclusion and equity in higher education [18]. Public policies have also been influenced by regional agreements. Specifically, the Declaration of the III Regional Conference on Higher Education for Latin America and the Caribbean held in 2018 assumed as a specific objective to ensure an inclusive, equitable and quality education. The historical, social and political diversity of national contexts has led to different initiatives in each country for the promotion of diversity. It must be also pointed that networks of institutions and students interested in the progressive consolidation of an inclusive higher education both in national areas (Colombian Network of Institutions of Higher Education for Disability or the Interuniversity Commission on Disability and Human Rights of Argentina, among others) and at a Latin American level (Latin American and Caribbean Interuniversity Network on Disability and Human Rights) have been created, making possible the opening of spaces for participation, research, exchange and dissemination of knowledge and inclusion practices [19]. Also, the boost of several socio-economic indicators at the start of the 21st century facilitated the incorporation of the indigenous culture in higher education institutions and the continuous pursuit for alternatives to achieve a higher calibre of education and the promotion of a fairer and more equal society [20, 21]. This positive context is conducive to greater commitment from countries to inclusivity within educational systems [22], whereby specific projects are conceived including the Measures for Social Inclusion and Equality in Higher Education Institutions in Latin America. An example of university social responsibility in Latin America is the Pathways program that serves indigenous students from Brazil, Chile, Mexico and Peru [23].

Meanwhile, public policies in Asia have been focused on boosting economic growth through higher education, seeking the most exceptional students to this effect. That said, students from ethnic minorities or rural areas who suffer socio-economic hardships are under-represented, thus challenging higher education institutions to tackle the issue of equality [24]. The state of affairs is different in Oceania. In particular, Australian universities have traditionally demonstrated a firm commitment to equality on the basis of national policies [25]. In 1988, the national higher education policy focused on equality following the publication of the White Paper *Higher Education*: *A Policy Statement*, explicitly stating the need to *"*change the balance of the student body to reflect more closely the structure and composition of society as a whole*"* [26]. On that basis, improvements were noted in the access and participation of several groups that to that point were under-represented, such as women, people with disabilities or those who speak a language other than English [27]. In 2009, in response to *The Bradley Review* [28], the Government drew up a new agenda with a focus on equal participation in higher education, especially from groups on which past action had been less effective, such as those with a low socio-economic status, indigenous peoples or residents in rural areas [29]. Subsequently, in 2015, the *Higher Education Participation Program* was approved, allocating funding to higher education institutions for the purpose of making important changes in the interest of achieving equality, diversity and inclusion.

Lastly, in Africa, the access to higher education is regarded as a privilege for few, thus reinforcing inequality in society with the exclusion of different students in terms of disability, ethnicity or race, culture, language or rural background [30]. In particular, less than 1% of people with a disability can access higher education [31]. Even in countries like South Africa, relevant race, gender and economic inequalities persist after a quarter of a century of anti-apartheid era [32].

### From economic logic to inclusion

Stepping back from a simplistic stance on diversity that gives a nod to the natural state of being and, thus, to differences between some individuals and others with regard to the above-mentioned characteristics, the concept of diversity embraces a broader spectrum that includes all of the ways in which people can differ [such as nationality, culture, religion, disability, sexual orientation, socio-economic status, language or learning styles] from an intersectional perspective [33]. However, Wentling and Palma-Rivas [34] sustain the idea that the concept of diversity can never capture the broad range of differences between some individuals and others. As a result, professionals should not solely focus on quantifying these factors, since diversity also encompasses many ways of grasping reality and acting accordingly.

One of the greatest concerns is the approach taken by higher education institutions to conceptualise the term 'diversity', since it conveys their stance and influences the type of actions that are taken. There are mainly two juxtaposing viewpoints: one based on economic logic, and the other on social justice.

From the economic perspective, it is believed that neoliberal politics have instilled a commercial or corporate approach into the culture of higher education institutions [35]. Universities have become an educational and social project whilst catering to economic needs and interests [36]. This university model calls for an elitist concept of knowledge under the logic of profitability and labour market demands. The term 'diversity' or 'diversity management' in higher education has been introduced from a commercial perspective based on the acknowledgment of difference or forms of representation, but without the implicit commitment to social justice [37]. Diversity is seen as a benefit for the national economy in the sense that it trains traditionally excluded groups [which may be women or ethnic minorities] in certain professions to later boost national productivity and competitiveness [38]. Initiatives based solely on increasing the heterogeneity of the student body and the celebration of diversity through ethnic festivals are examples of this stance that masks prevalent inequalities and discrimination [39], as is the case with the interaction of social class and culture [40] or the lack of attention to power and resources among racial/ethnic groups [41].

However, within the framework of social justice, the term diversity is linked to the solid commitment of the institution to address inequalities. The aim is to focus on identifying the attributes that lead to discrimination within higher education institutions and, consequently, developing timely actions to secure a level playing field with other people [42, 43]. From this standpoint, the term diversity is closely linked to equality. In this regard, Ahmed [44] believes that the term diversity has been used strategically by professionals as a solution to what has been known as "equity fatigue" or the vain attempt to wipe out inequalities. As such, it becomes associated with something new, once again highlighting the capacity for action by institutions to achieve equality.

Despite the fact that economic logic is the predominant rhetoric of diversity in higher education institutions [45], the link between the concept of diversity and equality and social justice could help facilitate progress in the exploration of exclusion processes intrinsic to academic institutions. That said, this endeavour does not automatically open up inclusive processes [46]. Hence the commitment to the paradigm of inclusion in response to diversity, which would comprise four fundamental issues [47]: it is a process; it focuses on identifying and breaking down barriers; it entails the presence, participation and progress of the entire student body; and it places a special focus on groups of students at risk of marginalisation, exclusion of underachievement.

On the other hand, the eagerness to secure diversity among the student body diverts attention from the heterogeneous profile of the faculty and administrative and service staff [48]. In other words, the complexity of the term diversity is intensified with the variety of people and spaces involved in higher education [49]. In this regard, the perspective of gender in both collectives is one of the most investigated topics in the research field [50].

#### The institutionalisation of diversity outreach in higher education

Focusing on internal initiatives and the social justice model, the success of the term diversity largely depends on the level of commitment or framework of action chosen by the decision makers within academic institutions [51, 52]. This, in turn, is subordinate to the utopian image they have of university [44]. Thus, it is pertinent to ask ourselves how we know if an institution is truly committed to diversity outreach within the framework of inclusive education, i.e., if it supports a discourse of transformation beyond a discourse of preservation [53].

In a pre-university educational context, the Index for Inclusion developed by professors Booth and Ainscow [47] is the self-assessment tool for inclusive processes in education centres with a greater international impact, although its validation in higher education has not yet been as successful as expected [54]. That said, there are higher education references in literature that propose dimensions or criteria to facilitate internal assessment and help plan initiatives that enable the institutional commitment to diversity outreach to materialise. The key differences between some proposals and others refer to the application, recipients, terminology used, and the context in which the criteria are identified, as seen in Table 1.

**Table 1:** Differences between the evaluation proposals of the institution's commitment to equality, diversity and inclusion.

	Gause, Dennison& Perrin, 2010 [49]	Ferreira, Vidal & Vieira, 2014 [55]	Michael, 2007 [56]	AAC&U, 2015 [13]	May & Bridger, 2010 [57]	NERCHE, 2016 [58]
APPLICATION						
Initiatives	✓	✓	✓		✓	
Evaluation instruments				✓		✓
RECIPIENTS						
Faculty staff	✓					
Students		✓		✓	✓	
University community			✓	✓		✓
TERMINOLOGY						
Equality	✓			✓		✓
Diversity	✓	✓	✓			✓
Inclusion	✓			✓	✓	✓

Insofar as the context is concerned, the criteria are established through meetings held by university committees [49], through those used by European and North American agencies that evaluate the quality of diversity [55], through literature review [56], through programs aimed at implementing inclusive policies and practices in higher education institutions [57], or by adapting other institutionalisation evaluation instruments [58].

Despite these differences, the studies mentioned share criteria that are conducive to institutionalisation. These include the addition of terminology to the mission or statement of the institution; the attribution of responsibility to someone who is a part of the senior management team within the institution; the creation of a formal body; the implementation of a strategic plan; integration across the organisational culture; the evaluation of progress and the adoption of improvement measures; the pairing with research agendas; specific initiatives linked to each one of the collectives comprising the university community; the curriculum, training and innovation.

According to Lloyd, Ordorika, & Rodríguez-Gómez [59], there are limitations and biases in the evaluation indicators of the international rankings. These are based on a university model: the elite research university. The student training, dissemination of culture, and attention to various responsibilities and commitments to society, are virtually absent from the rankings.

Social responsibility in diversity outreach with regard to these institutionalisation criteria is required from both entities with an average or low selective profile and those that are considered extremely selective or elite. Notably in relation to the latter, tensions emerge due to their determination to preserve an economic model based on meritocratic principles and competitiveness. Despite this, the moment diversity outreach becomes synonymous with the meanings of excellence, the more selective universities will likely be more welcoming of the idea to combine the commitment to disadvantaged students with the intent to preserve their prestigious status [60].

The study itself focuses on a sample of the best universities from around the world, according to the indicators used in developing what is known as the Shanghai ranking, and it is essentially aimed at exploring the processes to institutionalise diversity outreach in these universities. Thus, the basic research question addressed in the study is as follows: To what extent do the best world universities fulfil institutionalisation indicators in the area of diversity? Some expectancies could also be derived from the literature. If neoliberalism monopolises diversity practices, the best ranked universities and private institutions might fulfil more diversity indicators than low-ranked and public universities, respectively. A longer tradition in diversity advocacy, as is the case of the US, might also represent an advantage concerning the current diversity status of higher education institutions.

## Research methodology

### Design

A mixed method design was chosen, combining quantitative and qualitative approaches. On the one hand, a rationalist approach was selected with the intention to empirically quantify the degree of institutionalisation of diversity outreach through the perception of diversity outreach managers in higher education institutions in relation to a series of indicators, as well as the presence and main characteristics of these indicators in the information published on the websites of the selected universities.

The second part of the study was carried out within the qualitative paradigm. Through documentary analysis of the statements, the goals sought through the strategic diversity outreach plans and the definitions of diversity proposed by the higher education institutions, a qualitative content analysis was conducted using a thematic analytical strategy and inductive approach.

### Sample

The necessary sample size was set using population data contained in the *Academic Ranking of World Universities* (ARWU). This was the first global ranking system (2003), published since 2009 by the *Shanghai Ranking Consultancy*, an independent higher education body that is not answerable to any university or governmental organisation. More than 1200 higher education institutions are registered every year, although only the top 500 in the ranking are published (2017 edition). The ranking is based on six objective indicators: the number of people who have won Nobel prizes and *Fields* Medals, the number of highly cited researchers selected by Thomson Reuters, the number of articles published in Science and Nature journals, and the documents indexed in *Science Citation Index Expanded* and *Social Science Citation Index*, and per capita performance. Although any ranking system is biased—each one is based on specific criteria and interests, the ARWU was selected because of its particular relevance in the international arena, strongly influencing higher education evaluation policies in many countries [61].

Stratified sampling proportional to the size was carried out with help from the complex samples option on the SPSS (v23) *software*. The stratification criteria used included the section or range in which the university was placed in the ranking [the following sections are established in the ranking: 1–100, 101–150, 151–200, 201–300, 301–400, 401–500], the type of ownership [the universities were grouped together in public and private to simplify the number of strata, despite the diverse ownership of some of them: public universities that received private funding were classified as public and, vice versa, private universities that had public funding were classified as private] and the world regions [the regions defined were as follows: North America, Latin America, Europe, Africa, Asia and Oceania], resulting in 72 strata (6 sections or ranges x 2 types of ownership x 6 world regions). Meanwhile, in the application an initial percentage of 30% of surveyed units (universities) were selected to be included in the sample, a pragmatic criterion that did not hamper the analysis of a potentially large amount of information. The sample was later altered by targeting a range of eligible units according to the size of the universities (between 1 and 6). On the basis of the above criteria, the sample included 127 universities, which were considered representative of the population of 500 universities (95% confidence level and 7.5% margin of error).

Secondly, all the chief diversity and equality officers that were identified in the websites of the 127 institutions made up the survey-based sample. The search resulted in 78 diversity outreach officers or managers who were invited to fill in an online instrument.

### Tools and procedure

Two tools, one interactive and another non-interactive, were used to log the information on the institutionalisation indicators of diversity outreach:

1. e-Rubric to Evaluate the Institutionalisation of Diversity Outreach in Higher Education, targeting key informants by email. The aim of this method was to examine the perception of 78 diversity outreach managers identified on the university websites (those responsible for the diversity were informed in the email sent and the e-rubric of the nature, objectives, voluntariness of the participation and confidentiality of the data. Only if the respondents agreed to cooperate in the study in the terms proposed, they could proceed to answer the instrument. On the other hand, the Committee on Bioethics and Biosafety of the University of Cordoba, which confirms that all the ethical requirements have been respected, explicitly informs about the voluntariness in the participation, and endorses the anonymity of the data. This Committee gave its approval to the methodology used, although regulations that are in force in Spain do not require a specific approval of survey-based studies in the Social Sciences like the one reported here). The English version of the tool was used given that it is the most used language in the different professional and academic areas, as well as in global communication on the whole. The e-Rubric was designed over two phases:
  - Firstly, a list of 24 institutionalisation criteria was drawn up from literature and grouped into 4 facets: 2 general (philosophy and institutional policy, and institutionalisation strategies aimed at the university community) and 2 specific (institutionalisation strategies aimed at teaching and research staff, and institutionalisation strategies aimed at administrative managers within the institution). The evidence was drawn up in relation to the three levels established in the institutionalisation process of each indicator: absence, in process and consolidation.
  - Secondly, the e-Rubric content was validated by two diversity experts, one of whom holds a position of responsibility in this field in a university context. Both experts commented on the drafting of 7 indicators, but had no objections with regard to the number or content thereof, or to the facets or levels of institutionalisation. Lastly, the drafting of the above-mentioned indicators was improved (S1 Appendix).
2. Log form of Institutionalisation Indicators of Diversity Outreach in Higher Education (see the categories and indicators in S2 Appendix) It is applied to the content analysis of the websites for the formal diversity outreach bodies in the selected universities for the purpose of determining the presence and main characteristics of the previously established indicators. Websites are significant information sources about the commitment of higher education institutions to diversity, inclusion, equity and social justice [62, 63]. Even counting the form initially with the same indicators as the e-Rubric, 7 of these were eventually eliminated (institutional culture, institutional context, accreditation, study programs, innovation, research and resource management) due to a lack of relative information on the websites of the selected institutions.

### Data analysis

The information obtained with both tools was codified in a SPSS v.23 table for quantification purposes (S1 Dataset). The analyses carried out were descriptive, as well as inferential when determining the statistical differences in the institutionalisation indicators according to the ranking ranges, ownership and world region.

In relation to the qualitative study, when reviewing the theoretical framework, no precedents for the key categories or units of analysis in the studied field were identified with the possibility of being transferred to this study. Therefore, the first phase entailed objectively delimiting and defining the meaning of the units of analysis based on the information recorded on the Log Form of Institutionalisation Indicators of Diversity Outreach in Higher Education. Subsequently, the occurrence rate of the topic in the proposed categories was codified. The IT program used to carry out the analysis was MAXQDA 12.

## Results

**Fig 1:**
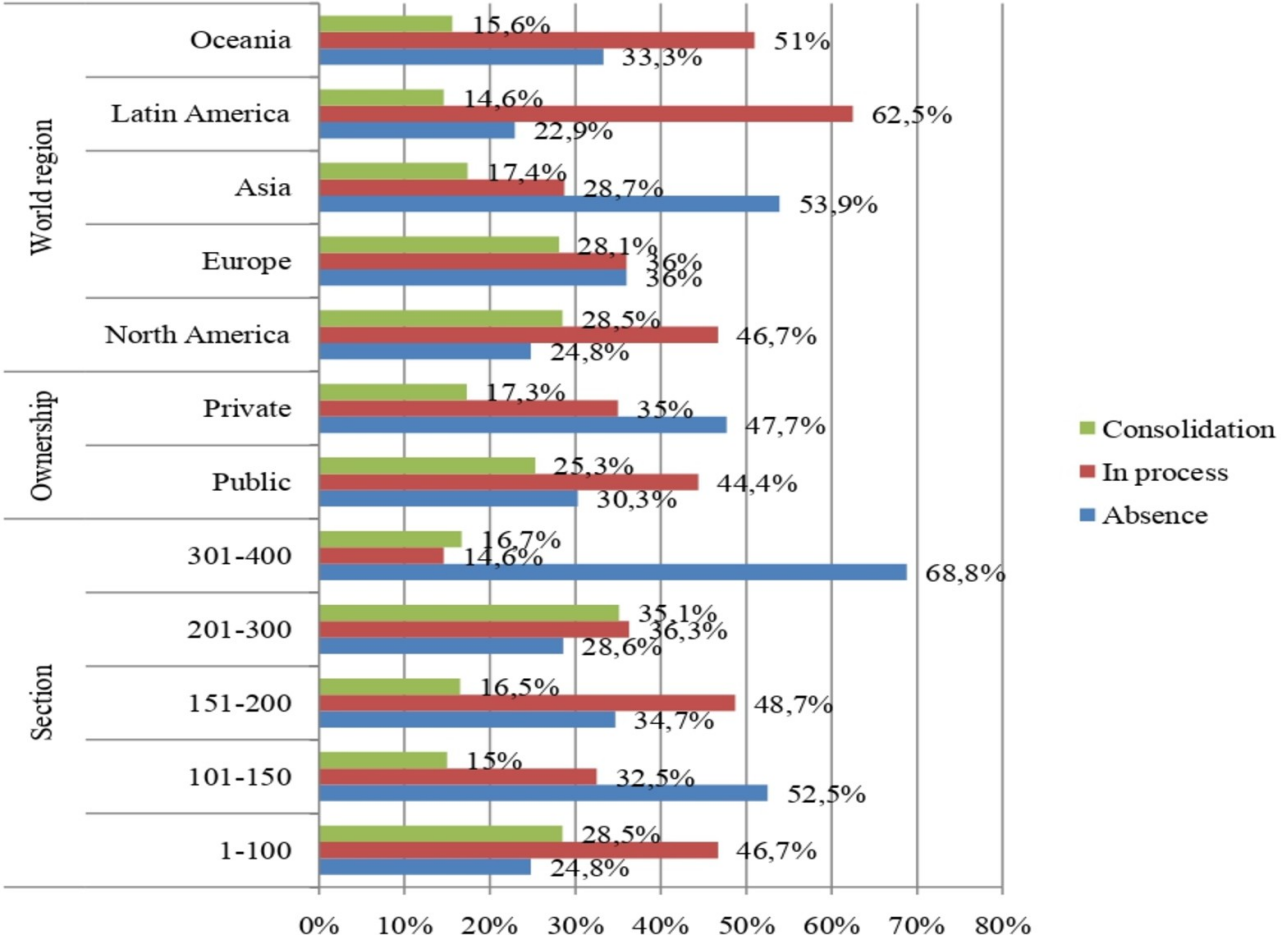
Relative frequencies of indicators at each institutionalisation level in relation to the section, ownership and world region.

Out of a total of 78 diversity outreach managers asked to take part, 29 responded to the e-Rubric (sections: N_section1_ = 5, N_section2_ = 5, N_section3_ = 10, N_section4_ = 7, N_section5_ = 2, N_section6_ = 0; ownership: N_public_ = 20, N_private_ = 9; and world regions: N_North America_ = 9, N_Europe_ = 7, N_Asia_ = 7, N_Latin America_ = 2, N_Oceania_ = 4, N_Africa_ = 0). The average frequency of indicators at each institutionalisation level established in the tool (absence, in process and consolidation) was 8.5, 9.9 and 5.5 respectively. Fig 1 separates these frequencies into each category in terms of region, ownership and ranking section.

**Fig 2:**
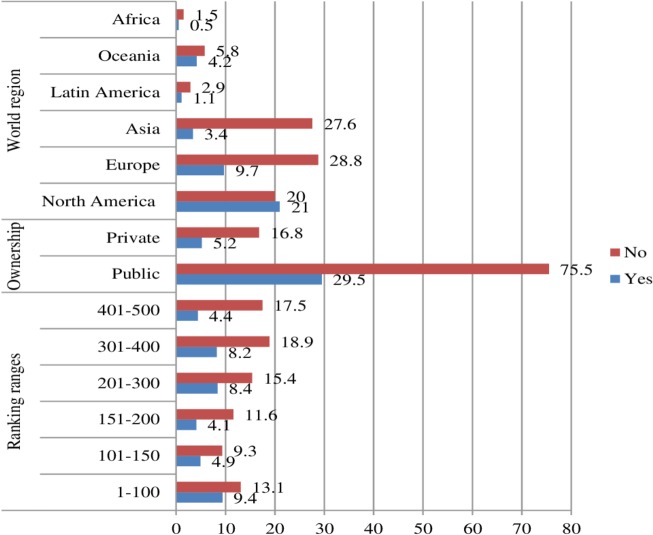
Average frequencies of universities that did or did not fulfil all institutionalisation indicators, in terms of ranking ranges, ownership and world region.

Coherent results were obtained through the Log Form of Institutionalisation Indicators of Diversity Outreach in Higher Education, applied to the content of institutional websites. The region of North America may be considered an exception, taking into account that the average frequencies of universities that fulfilled the indicators and those that did not, as depicted in Fig 2, are very similar. As a counterpoint, there are significant differences between these types of frequencies in the regions of Africa, Asia and Europe. This does not occur in Oceania, where the differences between some universities and others are minor. Meanwhile, regardless of the ownership of these universities, the majority of them did not fulfil the indicators. With regard to the sections or ranges in which the universities are placed in the ranking, the difference between the average frequencies of universities that fulfilled the indicators and those that did not is minor for universities positioned in the first range. Unexpectedly, universities positioned in the fourth and fifth range fulfilled more indicators than those positioned second and third.

In addition to measuring the average frequencies, the statistical significance of the differences was checked through Chi-Squared and Likelihood Ratio tests. Given that the analysed sample is representative of the population of universities on the ranking, the applied inferential tests help obtain information on the degree of homogeneity in fulfilling the indicators in the set of 500 universities.

The high number of expected frequencies lower than 5 called for the reformulation of the number of ranking ranges and world regions, in both cases dropping to three: 1–150, 151–300, 301–500, and North America, Europe and all other world regions [Oceania was not taken into account in this last category in order to avoid concealing a differentiated case study in relation to the other 3 regions included in the same level: Asia, Latin America and Africa] respectively. So, from the significance values listed in Table 2, it is clear that there are significant differences between universities according to the ranking ranges in the indicators concerning institutional statement, definition of diversity, formal centralised body, beneficiaries, quality assurance and/or institutional evaluation, and awards and acknowledgments. In these six indicators, the review of contingency tables helps prove that neither variable is independent, but instead that the frequency of universities that fulfil the indicators is always higher in the first range analysed (1–150), while the frequency of those that fail to fulfil them is always higher in the last range (301–500). Specifically, in the correlative order in which the six indicators were stated, the frequency of universities that fulfil them from the first range in comparison with the last is as follows: 26 *vs*. 20, 13 *vs*. 7, 14 *vs*. 5, 21 *vs*. 12, 7 *vs*. 1, and 11 *vs*. 9, respectively, and this is the case despite the fact that the total number of institutions was higher in the third range (n_1-150_ = 38, n_301-500_ = 48). On the other hand, this same comparison between the first range *vs*. the last one for universities that fail to fulfil the indicators was observed in the following pairs of frequency: 12 *vs*. 28, 25 *vs*. 41, 24 *vs*. 43, 17 *vs*. 36, 31 *vs*. 47, and 27 *vs*. 39, respectively.

**Table 2:** Error associated (*p*) with the values reported by the Chi-Squared and Likelihood Ratio tests in determining the statistical differences in fulfilment of institutionalisation indicators according to the ranking ranges, type of ownership and world region.

*Indicators*	Ranking ranges		Ownership		World region	
	*χ*^*2*^	LR	*χ*^*2*^ *with Yates correction*	LR	*χ*^*2*^	LR
Institutional statement	.036*	.034*	.910	.728	.000***	.000***
Strategic planning	.882	.883	.675	.500	.000***	.000***
Definition of diversity	.026*	.032*	.062	.042*	.000***	.000***
Administrative leadership	.582	.581	.364	.253	.000***	.000***
Formal centralised body	.009**	.010**	.563	.398	.000***	.000***
Beneficiaries	.017*	.016*	.995	.806	.000***	.000***
Leadership of the university community	.342	.367	1.000	.819	.291	.258
Support and guidance	.713	.717	.639	.458	.000***	.000***
Information and awareness	.870	.870	.021*	.008**	.000***	.000***
Training	.238	.240	.763	.576	.000***	.000***
Programs and initiatives	.088	.088	.706	.541	.000***	.000***
Quality assurance and/or institutional evaluation	.036*	.027*	1.000	.949	.000***	.000***
Institutional research	.099	.125	.067	.005**	.096	.071
Visibility of progress	.398	.402	1.000	.007**	.000***	.000***
Visible diversity	.726	.721	.535	.319	.001***	.000***
Collaboration with external entities	.737	.740	.603	.371	.000***	.000***
Awards and acknowledgments	.044*	.036*	.130	.040*	.000***	.000***

*p< .05.

**p< .01.

***p< .001.

With regard to ownership, significant differences were identified in both tests between public universities and private universities in one single indicator: information and awareness. In particular, the number of public universities that did not fulfil this indicator (n_no_ = 55) was only slightly higher that those who did (n_yes_ = 50). Meanwhile, the number of private universities that failed to fulfil the indicator (n_no_ = 18) significantly outnumbered the ones that did (n_yes_ = 4).

**Fig 3:**
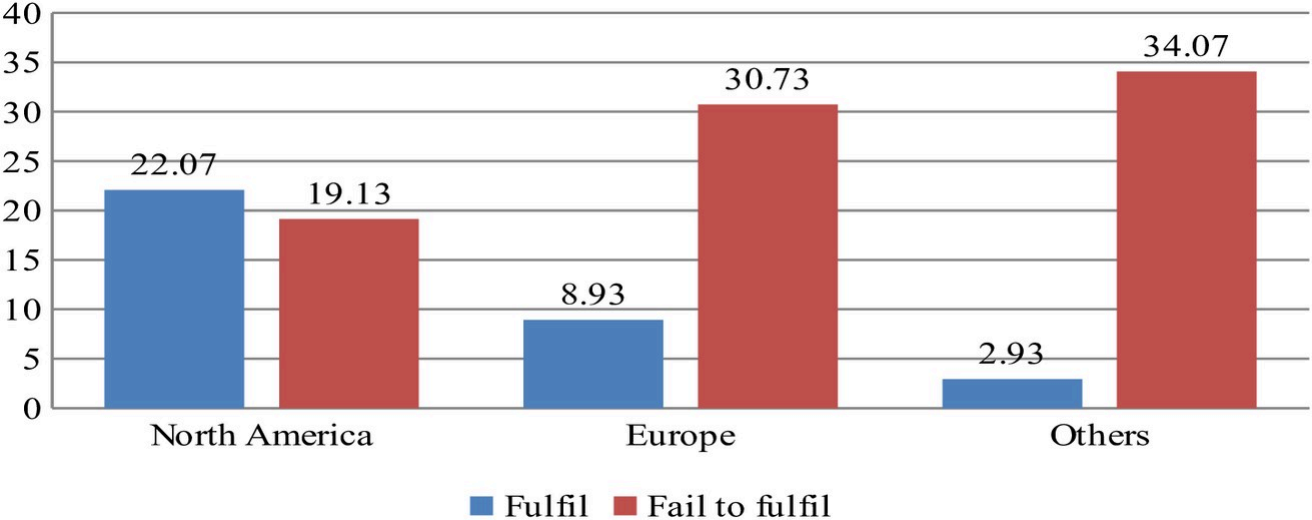
Areas and average frequencies of universities grouped together by large regions [North America, Europe, Others], which fulfil or fail to fulfil the 15 indicators in which statistical significance was reached in the Chi-Squared and Likelihood Ratio tests.

However, greater heterogeneity is observed in the geographical criterion. Only two of the institutionalisation indicators (leadership of the university community and institutional research) did not exhibit significant differences between universities according to the world region. In the other fifteen criteria, the North American region clearly exceeded Europe in the number of universities that fulfilled the indicators, while it fell well below the latter in the frequency of universities that failed to fulfil the indicators—the total number of universities in both geographical areas being roughly the same: n_North America_ = 41, n_Europe_ = 39—. On the other hand, the frequency of European universities that fulfilled the indicators was higher than the rest of the world, while the frequency is lower in terms of non-fulfilment, although the differential frequency was higher between North America and Europe than between the latter and the rest of the world. With the aim of specifying these statements, the average number was calculated for universities from each region that fulfilled or failed to fulfil the indicators in which statistical significance was reached, and the results outlined in Fig 3 were obtained.

S2 Appendix displays a summary of the comprehensive evidence [absolute and relative frequencies] regarding the institutionalisation status of diversity outreach in the selected universities, on the basis of the 17 indicators analysed on the institutional websites.

Out of a total of 127 universities, 58.3% have a formal centralised body for diversity outreach, while 61.4% have people holding decision-making positions in this area. More specifically, the diversity outreach manager is part of the senior management team in 20.5% of the institutions, while this person ranks lower in terms of responsibility in 26.8% of the universities.

With regard to the formal bodies, the beneficiaries of the actions of 49 of them are all of the collectives within the university community. That said, this is not adequately represented in the coordination team (13.5%). Half of the formal bodies have support and guidance systems in place, mainly the advisory council (25.7%), and with educational activities based on training programs and workshops as more common methods (29.7% and 28.4% respectively).

The most stable institutionalisation indicators in the formal bodies are those concerning information and awareness (73%) and support programs and initiatives for the university community (96%). Specifically, institutions mainly tend to hold events (43.2%) and organise conferences and talks (29.7%) as methods to raise awareness. Likewise, programs and initiatives aimed at members of the university community with disabilities or learning difficulties are common (47.3%), as are those targeting members who wish to issue an alert or complaint of discrimination or harassment (40.5%).

On the other hand, the least stable institutionalisation indicators in the formal bodies are connected to quality assurance or institutional evaluation (16.2%), institutional research (27%), progress visibility (27%), diversity in the demographic composition (29.7%), collaboration with external entities (25.7%) and awards and acknowledgments (31.1%). In relation to indicators of quality assurance or institutional evaluation, institutional research and awards and acknowledgments, the formal bodies have conducted research through surveys to determine the inclusive environment of the institution (12.2%), gender studies (9.5%), and they have given awards and acknowledgments to individuals with a solid commitment to diversity outreach (12.2%).

Alongside the simple quantification indicating that in half of the institutions there are references to diversity in the institutional statements (51.2%) and that less than half of them lack a strategic plan (26%) or a definition of diversity (19.7%), it is even more pertinent to determine the stance and lines of action taken by institutions that can be inferred on the basis of the qualitatively analysed data, assigning categories that, in turn, we have quantified.

**Fig 4:**
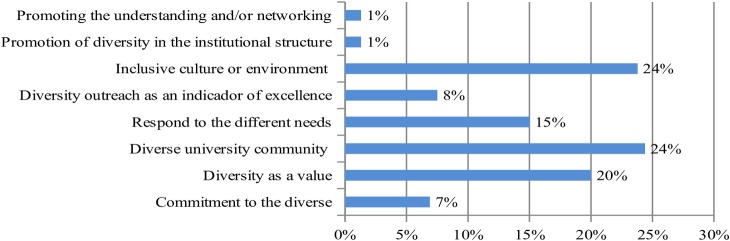
Distribution of frequency coding according to the institutional statement.

The aims set out by the institutions in their statements are primarily linked to the creation of a diverse university community and an inclusive culture or environment by celebrating diversity as a value, as shown in Fig 4.

**Fig 5:**
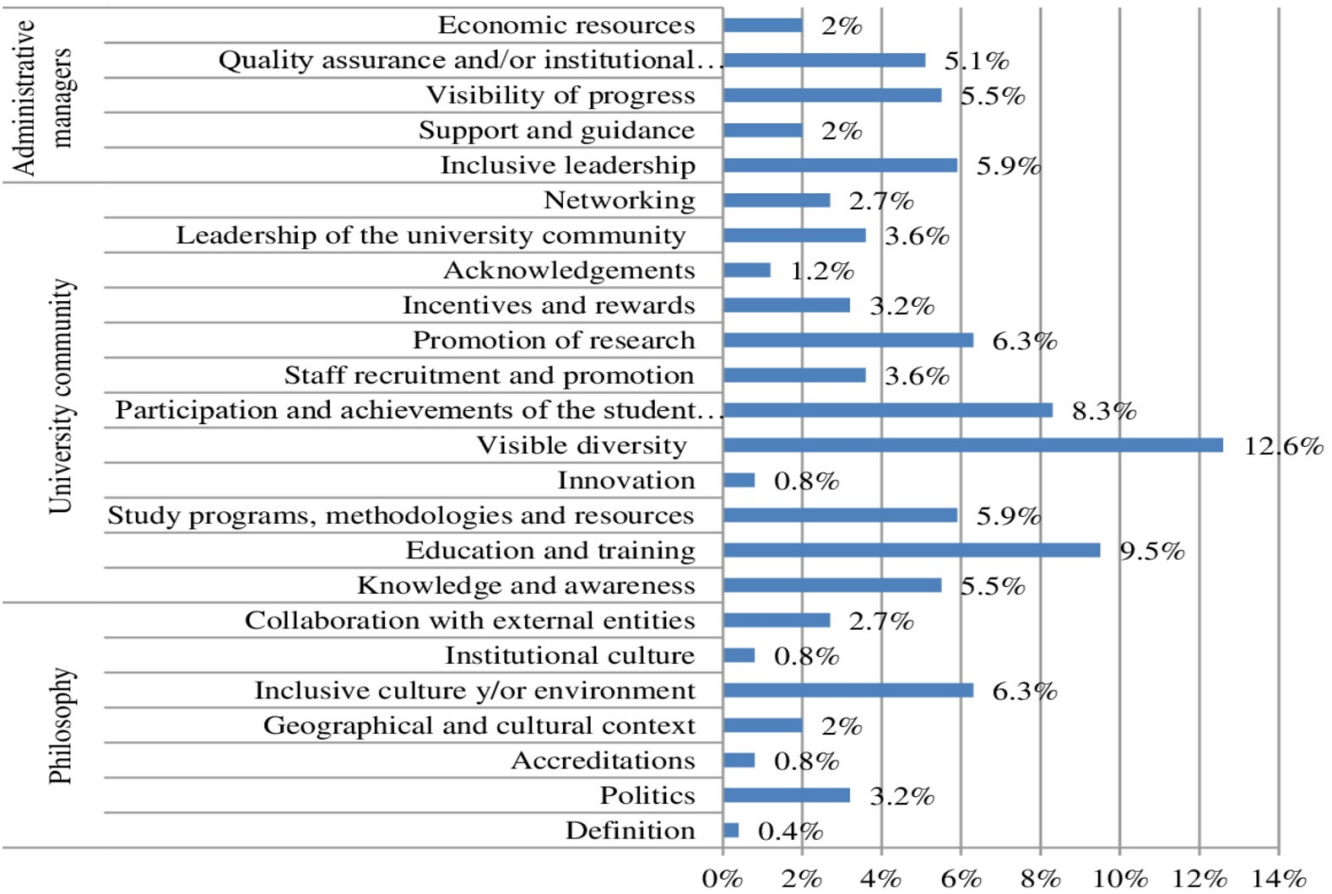
Distribution of frequency coding according to the goals of the strategic plans.

These results are linked to those obtained from the actions defined in strategic plan goals. In general terms, they are aimed at the university community (63.2%), administrative managers (20.6%) and the philosophy or approach taken by the institution regarding actions to address diversity (16.2%). More specifically, there is a higher number of actions linked to the creation of a diverse university community, education and training, as well as the participation and achievements of the student body, as seen in Fig 5.

**Fig 6:**
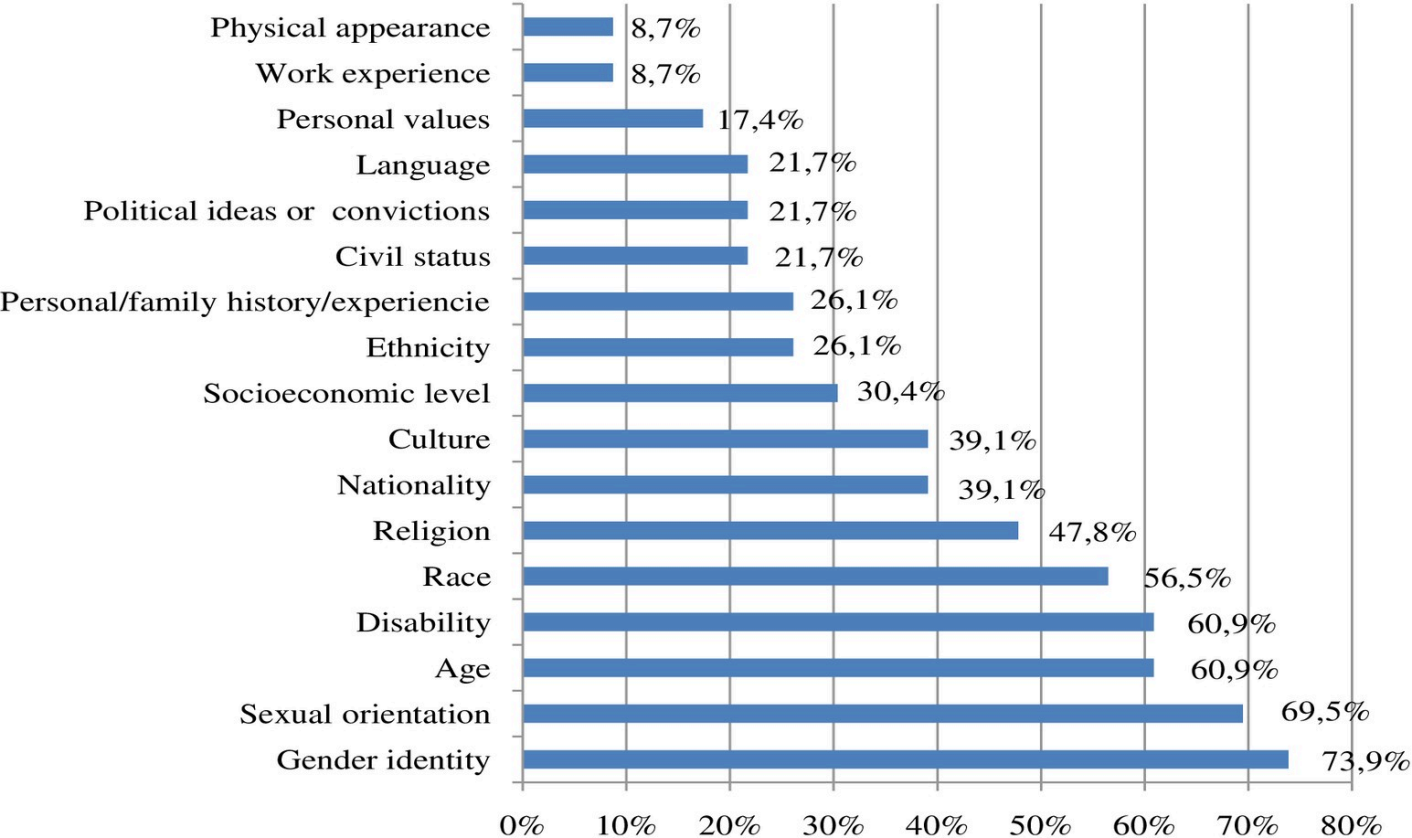
Distribution of frequency coding according to the concept of "diversity".

On the other hand, delving further into the concept of "diversity", the universities that have established a definition, have mainly interpreted the term as a "representation of differences" (73.9%). In much smaller percentages, they have clarified the meaning using terms of "inclusion" (21.7%), "respect" (13%) and "environment" (13%). There is a higher percentage of definitions that entail gender (73.9%), sexual diversity (69.5%), age (60.9%) and disability (60.9%) among the characteristics that make an individual different from another. To a lesser extent, they specify physical appearance (8.7%) and work experience (8.7%), as seen in Fig 6.

## Discussion and conclusions

The evidence gathered highlights that higher education institutions from the ARWU are at the beginning of process to institutionalise diversity outreach, in view of the low percentage obtained for the proposed indicators. Wide spread differences in the progress made in the institutionalisation process were not identified according to the type of ownership or ranking position of the institutions, although a certain degree of monopolization by neoliberalism on diversity practices might be inferred from differences found between first and last range institutions in six indicators. The fact that universities that perform better in the ARWU also stand out in some diversity indicators might provide some support to the market’s use of diversity measures [64]. Another factor that could have exerted some influence on the fulfilment of diversity indicators is the world region. Here, the historical background in the advocacy for civil rights and equal opportunities policies in world regions like North America [11, 29] has likely had a positive influence on the processes to consolidate diversity outreach.

As it was mentioned in the description of the sample, the ARWU was selected because of its influence on higher education evaluation policies in many countries. There are not many global rankings of significance—Hazelkorn [61] limits the number to ten—, three of them being considered as the most prominent: ARWU, Times Higher Education, and Quacquarelli Symonds [65]. Among the “big three”, ARWU outcomes seem to be more strongly predicted by objective research indicators, while the university ranks in the other two systems would be significantly predicted by expert-based reputation indicators [66]. Although objective indicators of ARWU could be taken as higher quality criteria, this system does not take into account the service mission of higher education, and it might partially explain the limited global percentage of consolidated institutional indicators that are fulfilled in the area of diversity, as well as the small number of differences that we found in fulfilment of indicators according to the ranking ranges. Since every ranking is biased, future research in this area should therefore use other global rankings and verify the prevalence of some diversity indicators in the top higher education institutions that we have found in the study reported here. Regional, national rankings and, even more interesting, meta-rankings as the one recently made by Luque-Martínez y Faraoni [67], should also be used to check the presence of diversity indicators in different ranges or in different clusters of universities. Multi-source evidence could provide a clearer picture about the relation of market- with social-based indicators, helping to interpret discrepant results when using different systems.Regarding the qualitative dimension of the study, in general terms, higher education institutions stipulate in their institutional statements and the goals of their strategic diversity outreach plans that one of the main aims is to increase the features of diversity in the demographic composition of the institution. Furthermore, this aim is connected to the language used in the definitions of diversity [37, 40]. The term is defined as a representation of differences. Thus, this study supports previous evidence that an economic approach to the conceptualisation might prevail, which acknowledges the differences or types of representation and attempts to boost the heterogeneity of the university community without making a social commitment to the inequalities [45]. In any case, it is apparent that the representation of a diverse community has yet to be achieved [68].

Not only the diverse demographic composition of universities, as well as institutional statements and definitions of diversity, could be at the service of middle-class whites and their structural power [69], but other indicators would also be drawn upon market-driven rationales—some of them related to differences found between ranking ranges—, thus reacting to the current phenomenon of massification in higher education [70]. This might be the case of the presence of a formal body in charge of coordinating diversity outreach efforts whose beneficiaries include the entire university community, the systematic and ongoing evaluations of diversity outreach initiatives, and formal mechanisms that encourage and acknowledge the involvement in activities linked to diversity outreach. All these factors make diversity visible and could be understood as publicity mechanisms aimed at gaining share in the global market of higher education. In fact, greater participation of different social groups is expanding in higher education, but a gap remains in educational and graduate outcomes, particularly in elite universities [71]. Nonetheless, each of the indicators deserve further research to explore how stakeholders give meaning to them in diverse institutions.

Meanwhile, the presence of a formal centralised body that coordinates diversity outreach endeavours in half of the higher education institutions could have also helped promote advocacy and awareness-raising actions, develop educational activities and, particularly, support programs and initiatives aimed at different groups of the university community. In the absence of a widespread commitment to a culture of inclusion, and even considering their market component, these three types of effects would support positive conclusions which are linked to the main lines of action outlined in the goals of the strategic plans, and which have possibly determined the increase and dissemination of best practices in regions such as Europe [16]. However, the institutions must continue to make progress on institutional research and evaluation processes, as well as on mechanisms that display the progress in order to make decisions based on objective data [42]. They should also collaborate with external social bodies that foster the enrichment and mutual recognition of the best practices developed in diversity outreach. All these suggestions could help approximate perspectives that are still far away from each other—market and diversity approaches—, as well as promote the convergence between universities and society.

The main restrictions found in the study are linked to language diversity and the content on institutional websites. A hypothesis on the reduced number of participants on the e-Rubric to evaluate the Institutionalisation of Diversity Outreach in Higher Education could be the difficulty experienced by some key informants in understanding the tool due to a lack of English, the language used for its dissemination considering it is the working language in the academic community. In addition, this difficulty based on a lack of knowledge of the language emerged in reading and registering the information found on the university websites, where the dominant language of the country where they are located is used. In order to overcome this difficulty, translators provided by the websites were of help. In addition to language challenges, another difficulty is linked to the use of institutional websites as sources of reliable, comprehensive and updated information, although they have been taken in other studies as significant information sources [62, 63]. A check of reliability would have involved conducting a survey that, in turn, would have been affected by an uncertain response rate.

In short, the universities should move forward in institutionalisation processes that help eliminate inequalities and promote social justice, even when excellence is booming. The solution may lie in mixing inclusion with excellence to create opportunities for personal, social and professional development for all, which calls for the institutions to adopt a solid commitment to the social responsibility they are assigned from political and civil bodies [52].

After verifying the initial stage that universities are at in the process to institutionalise diversity outreach, it is considered crucial to analyse the trends in this course of action. Perhaps now is the time to conduct longitudinal studies that help analyse the variations in institutionalisation indicators, and thus determine the evolution of the level of commitment made by universities to diversity outreach. It is an analysis that must be thoroughly contextualised, considering that the direction of higher education institutions is closely tied to politics, economy, ideologies and social attitudes, which are currently evolving faster than in eras past.

## Supporting information

S1 Dataset Data file. (XLSX)

S1 Appendix Facets and indicators of institutionalization. (PDF)

S2 Appendix Frequency and percentage of the selected universities, according to 17 institutionalisation indicators of diversity outreach. (PDF)
