# Supplementary material for: Evaluating the institutionalisation of diversity outreach in top universities worldwide
Source: PLoS One. 2019 Jul 24;14(7):e0219525. doi: 10.1371/journal.pone.0219525 (PMC6655642; doi:10.1371/journal.pone.0219525)
Supplement: S1 Appendix — (PDF) [file pone.0219525.s002.pdf]

S1 APPENDIX. Facets and indicators of institutionalisation

I. Philosophy and policy of higher education institutions in relation to diversity outreach
1. *Alignment with institutional statement*: the term diversity outreach is integrated in the institutional statement.
2. *Strategic planning*: the institution has an active plan that breaks down the actions geared towards diversity outreach.
3. *Definition of diversity*: the institution has an official and operational definition.
4. *Institutional culture*: there are initiatives that incorporate diversity into the organisational or operational structure of the university.
5. *Institutional context*: the diversity outreach actions are connected to the geographical, cultural and community context where the university is located.
6. *Accreditation*: the diversity outreach activities are taken into account in professional accreditation or promotion processes.
7. *Collaboration with external entities*: the institution promotes actions that demonstrate social commitment to areas of diversity (empirical research, projects coordinated with entities or bodies, etc.)
II. Institutionalisation strategies aimed at the university community (Teaching and Research Staff, student body, Administrative and Service staff, and management leaders)
8. *Information and awareness-raising of the university community on diversity outreach*: organised and systematic actions are carried out and aimed at the entire university community for the purpose of raising awareness and providing knowledge.
9. *Leadership in diversity within the university community*: there are opportunities to allow the university community to develop skills for taking on leadership roles to further the institutionalisation of diversity outreach.
10. *Incentive, rewards and recognition for the university community*: there are formal mechanisms that encourage the university community to take part in activities linked to diversity outreach, as well as its recognition.
11. *Visible diversity in the university community* (diversity is embodied in the demographic composition of the university community): mechanisms have been developed that facilitate access and retention of individuals belonging to under-represented groups.
12. *Support for the university community according to the type of needs* (visual impairment, physical disability, etc.) (accessibility of materials and spaces, sign language interpreter, curricular changes for students, etc.): there is enough support to guarantee participation in activities.
13. *Visibility of progress*: there is evidence of the professional development or learning achievements of under-represented groups in the university community.
III. Specific institutionalisation strategies of Teaching and Research Staff
14. *Study programs*: the institution encourages the reference to diversity in study programs.
15. *Training*: there is a selection of training activities for the faculty that offers a variety of teaching and learning approaches designed to respond to student diversity. This selection is part of the training plan.
16. *Innovation*: the innovative teaching practices related to diversity outreach are allocated enough resources.
17. *Research*: the institution implements initiatives to encourage research groups to include diversity in their plans.
IV. Specific institutionalisation strategies of administrative managers in the institution
18. *Administrative leadership*: the official responsibility to promote diversity outreach is assigned to someone belonging to the senior management team within the institution.

19. Support and guidance: there are support and guidance mechanisms in place for diversity outreach positions.
20.Formal body (service, office, unit, etc.): there is a formal body in charge of coordinating diversity outreach efforts.
21. Beneficiaries of the formal bodies: the beneficiaries of actions carried out in formal bodies include the entire university community.
22. *Resource management* (funding, resources, materials, staff, etc.): diversity outreach is allocated enough resources.
23. *Quality assurance/institutional evaluation* (through work groups or commissions): the institution conducts systematic and ongoing evaluations of diversity outreach initiatives.
24. *Institutional research*: the institution promotes studies that help develop diversity outreach initiatives based on research data.
