# Supplementary material for: Evaluating the institutionalisation of diversity outreach in top universities worldwide
Source: PLoS One. 2019 Jul 24;14(7):e0219525. doi: 10.1371/journal.pone.0219525 (PMC6655642; doi:10.1371/journal.pone.0219525)
Supplement: S2 Appendix — (PDF) [file pone.0219525.s003.pdf]

S2 APPENDIX. Frequency and percentage of the selected universities, according to 17 institutionalisation indicators of diversity outreach

INDICATOR	CATEGORIES	fa	fr
Reference made to diversity outreach in the institutional[1] statement	*Yes, a reference is made*	65	51.2%
	No reference is made	34	26.8%
	Unspecified (does not appear on the university's statement)	28	22%
Strategic planning for diversity outreach	*Yes*	33	26%
	No	89	70.1%
	Only of one specific aspect of diversity	5	3.9%
Definition of diversity	*Yes*	25	19.7%
	No	102	80.3%
Administrative leadership level[2] of diversity outreach	*Yes, it has*	78	61.4%
	• *Level 1* (*vice-president*, *vice-principal*, *vice-chancellor*, *vice-rector*, *vice-provost*, etc.)	26	20.5%
	• *Level 2* (*assistant principal*, *associate vice-chancellor*, *director, etc.)*	34	26.8%
	• *Others*: *academic lead*, *executive dean*, etc.	6	4.7%
	• *Position not specified*	12	9.4%

[1] There is heterogeneity in the term used by each university to convey such a formal statement. Other terms taken into account also include 'mission', 'vision', 'values', 'purpose of university', 'strategic goals', 'identity', etc.

[2] The organisational structure or leadership structure of higher education institutions is diverse. Efforts were made to homogenise the management levels of the selected universities in order to facilitate data analysis.

		N/A	49	38.6%
Organisational model of diversity outreach		*Yes, it has a formal body*	74	58.3%
		• *Centralised body*	39	21.9%
		• *Centralised body on which other services/departments/areas depend*	27	15.2%
		• *Several independent formal bodies*	8	3.9%
		No, it does not have a formal body	53	2.4%
Formal bodies	➢ Beneficiaries	*University community*	49	66.2%
		Focus on one specific aspect of diversity in the university community	4	5.4%
		Students	3	4.1%
		Focus on one specific aspect of diversity in students	10	1.4%
		Not specified	8	10.8%
	➢ Leadership of university community	*Yes*	10	13.5%
		No	64	86.5%
	➢ Support and guidance	*Yes*	37	50%
		• *Advisory council*	19	25.7%
		• *Technical committee*	15	20.3%
		• *Group of experts*	3	4.1%

		• *Work group*	8	10.8%
		• *Commission*	8	10.8%
		No	37	50%
	➢ Information and awareness	*Yes*	54	73%
		• *Workshops and meetings*	13	17.6%
		• *Festivities (special days, event weeks, commemorations, etc.)*	32	43.2%
		• *Guides or manuals*	14	18.9%
		• *Journals*	3	4.1%
		• *Forum*	11	14.9%
		• *Conferences and talks*	22	29.7%
		• *Awareness-raising campaigns*	8	10.8%
		• *Books, cinema, videos and exhibitions*	21	28.4%
		• *Leaflets, posters and newsletters*	14	18.9%
		• *Glossary*	3	4.1%
		No	20	27%
	➢ Training	*Yes*	41	55.4%
		• *Training courses*	17	23%
		• *Workshops*	21	28.4%

		• *Seminars*	7	9.5%
		• *Symposiums*	6	8.1%
		• *Education and training programs*	22	29.7%
		No	33	44.6%
	➢ Support programs and initiatives for the university community	*Yes*	71	96%
		• *Skills diversity (disability and learning difficulties)*	35	47.3%
		• *Gender diversity*	17	23%
		• *Racial diversity*	4	5.4%
		• *Ethnic-cultural diversity*	16	21.6%
		• *Sexual diversity (LGBT)*	10	13.5%
		• *Linguistic diversity*	4	5.4%
		• *Age diversity*	6	8.1%
		• *Economic diversity*	5	6.8%
		• *Religious diversity*	4	5.4%
		• *Alert or complaint of discrimination or harassment*	30	40.5%
		• *Transition to university*	6	8.1%
		• *Volunteering*	9	12.2%
		• *Creation of groups or networks for diversity work*	15	20.3%

		• *Dispute resolution. Mediation*	8	10.8%
		• *Tutoring, mentoring, etc.*	8	10.8%
		• *Employment (access, retention, work-life balance, etc.)*	10	13.5%
		No	3	4.1%
	➢ Quality assurance. Institutional evaluation	*Yes*	12	16.2%
		• *Inclusive environment survey*	9	12.2%
		• *Equality in employment survey*	3	4.1%
		No	42	56.8%
	➢ Institutional research	*Yes*	20	27%
		• *Gender studies*	7	9.5%
		• *Impact analysis of diversity and equality policies*	6	8.1%
		• *Studies on disability and learning difficulties*	4	5.4%
		• *Studies on access and retention of under-represented groups*	3	4.1%
		No	54	73%
	➢ Visibility of progress	*Yes (low frequency reports or statements: annual, bi-annual)*	20	27%
		No	54	73%
	➢ Visible diversity	*Yes*	22	29.7%
		No	52	70.3%

	➢ Collaboration with external bodies	*Yes*	19	25.7%
		No	55	74.3%
	➢ Awards and acknowledgement	*Yes*	23	31.1%
		• *Excellence*	5	6.8%
		• *Innovative programs and initiatives*	4	5.4%
		• *Commitment*	9	12.2%
		• *Research, dissemination and awareness*	3	4.1%
		No	51	68.9%
